# Proteomic dataset comparing strains of *Leptospira borgpetersenii* serovar Hardjo cultured at different temperatures

**DOI:** 10.1016/j.dib.2022.108713

**Published:** 2022-10-29

**Authors:** Ellie J. Putz, Luis G.V. Fernandes, Darrell O. Bayles, John D. Lippolis, Jarlath E. Nally

**Affiliations:** a Agriculture Research Service, National Animal Disease Center, 1920 Dayton Ave, Ames, IA 50010 USA; b Laboratorio de Desenvolvimento de Vacinas, Instituto Butantan, Avenida Vital Brasil, 1500, Sao Paulo, SP 05503-000, Brasil

**Keywords:** Leptospirosis, *Leptospira*, Proteomics, HaN media, Hardjo

## Abstract

Leptospirosis is a global zoonotic bacterial disease which is a threat for humans and most mammals. Bacterin vaccines for leptospirosis are available however they are severely limited in cross protection between serogroups. *Leptospira* typically colonize the kidneys of reservoir hosts where they are subsequently shed in the urine and persist in the environment and can thus be indirectly or directly transmitted to incidental hosts. *Leptospira borgpetersenii* serovar Hardjo is the primary cause of leptospirosis in cattle which can result in abortion, unhealthy calves, and rebreed problems. This dataset comprises proteomic profiles of four strains of *L. borgpetersenii* serovar Hardjo propagated at the routinely utilized culture temperature of 29 °C, and a newly achieved culture temperature of 37 °C, which more closely emulates the temperature of an infected host. The strains analyzed include JB197 (established strain that causes Hardjo atypical acute disease in the hamster model of leptospirosis), HB203 (established strain, causes typical chronic disease in hamsters), as well as TC129 and TC273 (recently isolated strains from the central United States). Differential expression profiles were detected not only between strains but also within strains between culture temperatures. Mass spectrometry data are available via ProteomeXchange with identifier PXD032831.

**Specifications Table**

**Table:** 

Subject	Microbiology: Bacteriology
Specific subject area	Proteomic profile comparisons of strains of *Leptospira borgpetersenii*
Type of data	Table
How the data were acquired	Mass spectrometry and peptide chromatography utilized the Thermo Dionex UltiMate 3000 RSLCnano system; Mass spectrometry raw data files were processed with the MaxQuant software, and additional analysis was conducted with the Perseus software.
Data format	Analyzed. The mass spectrometry proteomics data have been deposited to the ProteomeXchange Consortium via the PRIDE partner repository with the dataset identifier PXD032831. https://www.ebi.ac.uk/pride/archive/projects/PXD032831
Description of data collection	JB197, HB203, TC129, and TC273 strains of L. *borgpetersenii* serovar Hardjo were cultured at 29 °C and 37 °C. Whole cell lysate proteomic profiles were evaluated by mass spectrometry and analyzed with MaxQuant and Perseus software.
Data source location	*USDA Agricultural Research Service,* *National Animal Disease Center* *Ames, IA, USA*
Data accessibility	Repository name: PRIDE Proteomics Identifications Database Data identification number: PXD032831. https://www.ebi.ac.uk/pride/archive/projects/PXD032831
Related research article	E.J. Putz, L.G.V Fernandes, S. Sivasankaran, D.O. Bayles, D.P. Alt, J.D. Lippolis, J.E. Nally, Some like it hot, some like it cold; Proteome comparison of *Leptospira borgpetersenii* serovar Hardjo strains propagated at different temperatures, Journal of Proteomics 262 (2022) 104,602. https://doi.org/10.1016/j.jprot.2022.104602

## Value of the Data

• These data illustrate distinct proteomic profiles associated with highly similar genomic strains of *L. borgpetersenii* serovar Hardjo and demonstrate the role of temperature, as encountered during host infection, in regulating protein expression.
• These data illustrate strain to strain variation in protein expression and provide reference comparisons for additional strains and serovars of *L. borgpetersenii*, as well as additional species of *Leptospira*.
• Understanding strain to strain variation is critical to bacterin vaccine design and efficacy.
• The proteomic expression information provided here serves as a reference for the behavior of *L. borgpetersenii* serovar Hardjo strains in response to change in temperatures encountered during host infection.

## 1 Data Description

Data describes whole cell proteomics of four strains of *L. borgpetersenii* serovar Hardjo (JB197, HB203, TC129, TC273) cultured at 29 °C and 37 °C. Raw mass spectrometry data was analyzed by MaxQuant and Perseus for differential protein expression of interest. The 50 most highly expressed proteins across all strains and conditions are shown in Table 1. Full data and specific contrasts are available in Supplementary Table 1 (includes all output data from Perseus DE analysis. Strain JB197 at 29 °C was used as the reference condition).

**Table 1:** The fifty most highly expressed proteins across all strains and conditions based on abundance scores generated by MaxQuant and Perseus analysis. Proteins identified by UniProt ID and Fasta annotation. Shown are average abundance score by strain and condition combination over four biological replicates.

Protein IDs	Fasta headers	JB197 29 °C	JB197 37 °C	HB203 29 °C	HB203 37 °C	TC129 29 °C	TC129 37 °C	TC273 29 °C	TC273 37 °C
Q6J0P4	Immunodominant outer membrane protein LipL32	33.33	32.74	33.32	32.85	33.06	32.89	32.88	32.84
P61438	60 kDa chaperonin	31.40	32.72	31.63	32.69	32.08	32.64	31.68	32.74
Q04PT6	Elongation factor Tu	31.59	32.13	31.93	32.39	32.42	32.67	32.16	32.28
Q04UY1	LigB lipoprotein	31.48	31.50	34.16	31.83	29.08	29.21	33.16	31.87
Q04QD6	Electron transfer flavoprotein, alpha subunit	31.28	31.54	31.18	31.47	31.52	31.62	31.48	31.48
Q054E1	DNA-directed RNA polymerase subunit beta	31.56	31.18	31.28	31.40	31.50	31.48	31.57	31.44
M6XN61	Flagellin	31.78	31.70	31.42	31.30	31.28	31.32	31.30	31.27
P61443	Chaperone protein DnaK	29.96	32.05	29.96	32.03	30.78	32.18	30.61	31.89
Q04PQ5	Uncharacterized protein	31.31	31.02	31.08	30.63	31.11	30.90	31.39	30.79
Q8F0S2	DNA-directed RNA polymerase subunit beta n	31.10	30.70	30.79	30.97	31.08	31.07	31.08	30.96
Q04RL2	Uncharacterized protein	31.23	30.98	30.88	30.94	30.81	30.89	30.80	30.85
Q04TE2	Cysteine synthase	30.56	30.46	31.03	30.95	30.76	31.12	31.08	31.12
Q04S18	ATP synthase subunit beta	31.17	30.45	30.51	30.65	31.32	31.28	30.95	30.69
M6UPA6	Uncharacterized protein	31.06	31.40	30.38	30.69	30.87	30.53	30.84	30.61
P61436	10 kDa chaperonin	29.63	31.12	30.29	31.25	30.96	31.12	30.54	31.24
A0A1B9FII9	ArsR family transcriptional regulator	31.30	31.17	30.72	30.66	30.50	30.77	30.39	30.52
A0A0E3AZB2	LysM domain-containing protein	31.08	30.85	30.98	30.19	30.69	30.51	30.75	30.25
M6A6Z4	4Fe-4S dicluster domain protein	30.84	30.52	30.38	30.54	30.28	30.71	30.35	30.61
Q04UP0	Transcription elongation factor GreA	30.42	30.50	30.20	30.51	30.66	30.67	30.73	30.50
M3ERF2	Glutamine synthetase, type I	30.83	30.48	30.69	30.00	31.10	30.13	30.57	30.11
Q72MM7	Uncharacterized protein	30.68	30.42	30.47	30.38	30.34	30.13	30.32	30.25
Q04PW5	DNA-directed RNA polymerase subunit alpha	30.52	30.14	30.35	30.33	30.41	30.38	30.43	30.38
M6AK61	Redoxin	30.13	30.24	30.70	29.95	31.16	30.31	30.30	30.13
Q04Y01	Elongation factor G	29.88	30.06	29.91	30.23	30.86	30.60	30.48	30.04
Q04UP1	Endoflagellar filament sheath protein	30.66	30.23	30.23	30.32	29.82	30.24	30.13	30.26
Q04NS0	HSP90	29.46	30.67	28.90	30.74	29.96	30.90	29.96	30.80
A0A1D7USI2	OmpA-like domain-containing protein	30.10	30.40	30.11	29.94	30.48	30.18	30.06	29.90
Q04P78	ABC_transp_aux domain-containing	30.44	30.65	30.00	30.34	29.33	30.19	29.71	30.40
Q04VX0	Isocitrate dehydrogenase [NADP]	29.33	29.66	29.93	30.54	30.28	30.51	30.16	30.57
Q04QD7	Electron transfer flavoprotein, beta subunit	29.90	30.09	29.85	30.11	30.32	30.21	30.20	30.16
A0A1D7V0C6	TPR_REGION domain-containing protein	31.03	30.45	30.64	29.08	30.57	28.91	30.59	29.28
Q72SY1	ATP synthase subunit alpha	30.31	29.56	29.73	29.82	30.61	30.33	30.06	29.80
M3GL54	Methyl-accepting chemotaxis protein signaling domain protein	30.32	29.54	30.94	30.11	29.39	30.18	29.58	29.68
M6CD43	Uncharacterized protein	29.66	28.40	30.97	29.23	30.76	29.71	30.72	29.55
A0A0C5 × 1S3	Cytoplasmic membrane lipoprotein LipL31	30.08	29.83	29.79	29.87	29.84	30.07	29.65	29.84
Q04TN1	Methylase/methyltransferase	29.93	30.20	29.72	29.56	30.00	29.49	30.07	29.60
Q04SY8	Nuclease S1	29.48	31.00	30.36	29.89	28.12	29.70	29.79	30.11
Q04QS3	Succinate–CoA ligase [ADP-forming] subunit beta	29.64	29.80	29.66	29.64	30.08	30.03	30.00	29.58
V6IAH4	Acetyl-CoA C-acyltransferase n	29.63	29.23	29.80	29.54	30.19	30.26	29.69	29.70
Q6GXD1	Transmembrane outer membrane protein	30.87	30.50	30.06	28.45	30.24	29.31	29.54	28.84
Q72VT0	Phosphoenolpyruvate carboxykinase (ATP)	29.29	29.33	29.37	29.66	30.02	30.01	29.98	29.78
Q04PG8	Cytochrome c oxidase subunit 2	29.81	29.45	29.55	29.73	29.68	29.57	29.86	29.78
Q04QF8	Uncharacterized protein	29.99	29.86	30.13	29.18	29.51	29.59	29.73	29.31
Q04ZJ9	Polyribonucleotide nucleotidyltransferase	29.54	29.21	29.81	29.50	29.81	29.69	30.00	29.52
A0A540UB21	*Re*/Si-specific NAD(P)(+) transhydrogenase subunit alpha	29.80	29.69	29.16	29.70	29.70	29.82	29.48	29.68
Q04P68	3-hydroxyacyl-CoA dehydrogenase	29.29	29.22	29.03	29.62	30.23	30.45	29.44	29.65
Q04W30	Membrane protein insertase YidC	29.81	29.44	29.29	29.45	30.00	29.81	29.72	29.34
Q04U49	Chemotaxis protein histidine kinase	29.72	29.51	30.37	29.72	28.70	29.56	29.36	29.57
A0A2H1XGQ9	Fructose-bisphosphate aldolase class I	29.35	29.36	29.32	29.65	29.45	29.87	29.45	29.70
Q04NN6	Adenosylhomocysteinase	29.04	29.27	29.07	29.68	29.88	29.72	29.60	29.87

## 2 Experimental Design, Materials and Methods

### 2.1 Culture of Leptospira Strains

*L. borgpetersenii* serovar Hardjo-bovis strains JB197 (accession: PRJNA16148), HB203 (accession: PRJNA384237), TC129, and TC273 (accession: PRJNA759631, [1]) were cultured in HAN media at 29 °C and 37 °C respectively [2]. At mid-late log phase, bacteria were centrifuged (4 °C, 10,000 x *g*, 30 min), and the pellet was washed with cold TE buffer twice, and stored at -80 °C. Four biological replicates per each condition were prepared for proteomics.

### 2.2 Trypsin Digestion

Samples were digested according to manufacturer's directions with Trypsin/Lys-C Mix (Promega Corporation, Madison, WI) as previous reported [3]. After digestion, samples were run through a desalting Pierce C18 spin column (Thermo Fisher Scientific, West Palm Beach, FL) according to manufacturer's instructions. After drying by vacuum (Speedvac), samples were resuspended in 95% H_2_O 5% acetonitrile and 0.1% formic acid.

### 2.3 Mass Spectrometry

Mass spectrometry and peptide chromatography were completed as done previously [4]. The Thermo Dionex UltiMate 3000 RSLCnano system (Thermo Fisher Scientific, FL, USA) instrument was utilized for peptide chromatography. Chromatography was accomplished with a gradient of buffer A (95% H2O: 5% acetonitrile and 0.1% formic acid) and buffer B (5% H2O: 95% acetonitrile and 0.1% formic acid) over an Acclaim PepMap 100 C18 column. The LC was connected to a Nanospray Ion Source (Thermo Fisher Scientific, FL, USA) on a LTQ OrbiTrap Velos Pro (Thermo Fisher Scientific, FL, USA) mass spectrophotometer. The mass spectrometer used these settings: MSMS scanning resolution was 15,000, the 400 to 2000 *m/z* MS resolution was 30,000, the repeated mass duration exclusion was 1 min, and CID activation was utilized (normalized with a collision energy of 35). A signal minimum of 5000 was required for the MS Top 10. The *L. borgpetersenii* serovar Hardjo-Bovis database was downloaded from UniProt (August 2020). The mass spectrometry proteomics data have been deposited to the ProteomeXchange Consortium via the PRIDE [5] partner repository with the dataset identifier PXD032831.

### 2.4 Analysis of Proteomic Data

Raw mass spectrometry data went through initial processing by MaxQuant (Version 1.6.7.0) [6] followed by subsequent Perseus analysis [7] as reported previously [3]. MaxQuant fixed modifications consisted of Carbamidomethyl (C) and variable modifications included acetyl (Protein N-term), deamidation (NQ), oxidation (M), and Phosphorylation (STY). In Perseus, data was log2 transformed, single peptide identified proteins were removed, and differential expression contrasts were calculated and reported in completion in Supplementary Table 1. For summary of the fifty most highly expressed proteins across strain and temperature shown in Table 1, all abundance scores generated in MaxQuant and Perseus were summed and sorted to identify the fifty most abundant proteins. Table one shows the average abundance score per strain and temperature condition among four biological replicates.

### 2.5 Statistical Analysis

Perseus software was used to analyze proteomics data [7]. Differential expression utilized a P-value < 0.01 for significant differences.

## CRediT authorship contribution statement

**Ellie J. Putz:** Conceptualization, Methodology, Formal analysis, Investigation, Visualization, Writing – original draft, Writing – review & editing. **Luis G.V. Fernandes:** Methodology, Formal analysis, Data curation, Writing – review & editing. **Darrell O. Bayles:** Methodology, Formal analysis, Validation, Writing – review & editing. **John D. Lippolis:** Conceptualization, Methodology, Formal analysis, Writing – review & editing. **Jarlath E. Nally:** Conceptualization, Validation, Formal analysis, Investigation, Data curation, Supervision, Writing – original draft, Writing – review & editing.

## Declaration of Competing Interest

The authors declare that they have no known competing financial interests or personal relationships that could have appeared to influence the work reported in this paper.

## Data Availability

Proteome comparison of *Leptospira borgpetersenii* serovar Hardjo strains cultured at different temperatures (Original data) (PRIDE Proteomics Identification Database).

## Appendix Supplementary materials

Image, application 1
